# Annexin-A1 promotes RIG-I-dependent signaling and apoptosis via regulation of the IRF3–IFNAR–STAT1–IFIT1 pathway in A549 lung epithelial cells

**DOI:** 10.1038/s41419-020-2625-7

**Published:** 2020-06-15

**Authors:** Gracemary L. R. Yap, Karishma Sachaphibulkij, Sok Lin Foo, Jianzhou Cui, Anna-Marie Fairhurst, Lina H. K. Lim

**Affiliations:** 1 Department of Physiology, Yong Loo Lin School of Medicine, National University of Singapore, Singapore, Singapore; 2 NUS Immunology Program, Life Sciences Institute, National University of Singapore, Singapore, Singapore; 3 NUS Graduate School for Integrative Sciences and Engineering, National University of Singapore, Singapore, Singapore; 4 Institute for Molecular and Cellular Biology, A-STAR, Singapore, Singapore

**Keywords:** Infection, RIG-I-like receptors

## Abstract

Within the last century, millions of lives have been lost to the four major Influenza pandemics. These influenza pandemics were all caused by Influenza Type A viruses (IAV) through their ability to undergo antigenic drifts and shifts. A greater understanding of IAV and host–pathogen interactions is required to develop effective therapeutics against future outbreaks. Annexin A1 (ANXA1) is a phospholipid binding, calcium-dependent protein known to play essential roles in multiple cellular functions including inflammation, proliferation, migration, and apoptosis. ANXA1 was previously shown to enhance apoptosis after IAV infection. The current study explores the role of ANXA1 in IAV infection of A549 lung epithelial cells further in the context of RIG-I-dependent signaling using A549 and Crispr/Cas9 ANXA1 deleted (A549∆ANXA1) cells. ANXA1 was found to enhance the expression of a cytoplasmic RNA sensor, RIG-I basally and post-infection. RIG-I activation by 5′ppp-RNA in A549 lung epithelial cell induces apoptotic cell death, which is inhibited when ANXA1 is deleted, and reversed when ANXA1 is re-expressed. RIG-I activation by 5′ppp-RNA stimulates the production of IFNβ from lung epithelial cells to the same extent as monocytic cells, albeit very late after infection at 48–72 h, through IRF3 and STAT1 activation. ANXA1 deletion delays the phosphorylation of IRF3 and STAT1, leading to lower expression of interferon-stimulated genes, such as IFIT1, and silencing IFIT1 inhibited RIG-I-induced cell death. In all, these results suggest that ANXA1 plays a regulatory role in RIG-I signaling and cell death in A549 lung epithelial cells.

## Introduction

Rig-I like receptors (RLRs) are cytosolic RNA sensors that belong to a family of DExD/H box RNA helicases, and they have the capacity to hydrolyze ATP. Presently, there are three known RLRs which include RIG-I (retinoic acid-inducible gene I, also known as RIG-I), melanoma differentiation-associated gene 5 (MDA5), and Laboratory of genetics and physiology 2 (LG P2)^1^. Upon binding of RLRs to their respective agonists, this triggers the activation of anti-viral responses in the cell. For RIG-I, the binding of the dsRNA with a 5′ppp-RNA motif results in a conformational change of RIG-I, a process dependent on ATP and allows oligomerization of RIG-I to form a tetramer^2^. The RIG-I tetramer forms a scaffold structure which allows for the binding to mitochondrial antiviral-signaling protein (MAVS), an adaptor protein present on the mitochondrial membrane^3^. RIG-I plays an essential role in IFN induction during RNA virus infections of non-pDC cell-types. Mice that are deficient in MDA5 and RIG-I are more susceptible to RNA viruses^4,5^. Ubiquitously expressed RIG-I can be detected in all cell types including tumor cells. However, the type of RIG-I-induced responses is different between cells. Normal healthy cells such as melanocytes and fibroblasts are quite resistant to RIG-I-induced apoptosis. However, tumor cells exhibit highly susceptible RIG-I-induced cell death^6,7^.

Annexin-1 (ANXA1) is a 37 kDa anti-inflammatory protein, and it is the first member in the annexin superfamily to be discovered. The annexin superfamily consists of 13 members and all bind to phospholipids in a calcium-dependent manner^8^. This protein superfamily is termed as Annexins due to their characteristic of annexing to membrane proteins^9^. As ANXA1 was first discovered to be anti-inflammatory, the role of ANXA1 as an anti-inflammatory and pro-resolving mediator of inflammation has become increasingly established^10,11^. In addition to its role in inflammation, ANXA1 also regulates apoptosis^12^.

We previously reported the roles of ANXA1 in the context of H1N1 influenza infection in both immune and lung epithelial cells^13^. The presence of ANXA1 promotes viral entry by influencing viral binding to target cells, enhances viral replication upon infection and promotes the trafficking of the virus to the nucleus. ANXA1^−/−^ mice were shown to have lower viral titers compared to wild-type mice and displayed increased survival upon infection of the H1N1 strain. In addition, the presence of ANXA1 was also found to enhance the induction of apoptotic cell death upon H1N1 infection of both monocytes and lung epithelial cells^13^. As H1N1 influenza virus is a single-stranded negative RNA virus, RNA sensors present in the cell are of particular importance. With a distinct focus of ANXA1’s role in TLR3^14^ signaling in past publications, this study now aims to discover and understand the role of ANXA1 in the activation and signaling of the cytoplasmic RNA sensor, RIG-I in the context of IAV infection-induced cell death in lung epithelial cells.

## Results

To study the role of ANXA1 in the context of H1N1 infection, ANXA1 was deleted from A549 cells using CRISPR-Cas9 technology. SgRNA were custom designed by and obtained from Horizon Discovery. Successful clones of A549 deficient in ANXA1 were selected from the gRNAs encoded by Plasmid 2 and Plasmid 4. Cell clones with less or no ANXA1 protein expressed, #2C and #4D, were further expanded and protein levels were determined by western blot (Supplementary Fig. 1A). To confirm that ANXA1 has been successfully deleted in A549∆ANXA1 cell line, Sanger sequencing was performed. The sequence targeted by sgRNA #4 in the ANXA1 gene was amplified using PCR and the product from Sanger sequencing revealed a single base pair insertion which is three-base pairs downstream of the protospacer adjacent motif (PAM). The human ANXA1 protein consists of four domains and the sgRNA #4 targets a sequence within the the first domain (Supplementary Fig. 1B). The frameshift mutation due to the single base pair insertion results in the formation of a truncated ANXA1 protein (Supplementary Fig. 1C). Furthermore, to ensure that there are no off-target effects, an online tool COSMID^15^ was used and no off-target mutations were found in any off-target sites (data not included). Henceforth, A549 ∆ANXA1 clone #4D was used in all subsequent experiments.

**Fig. 1:**
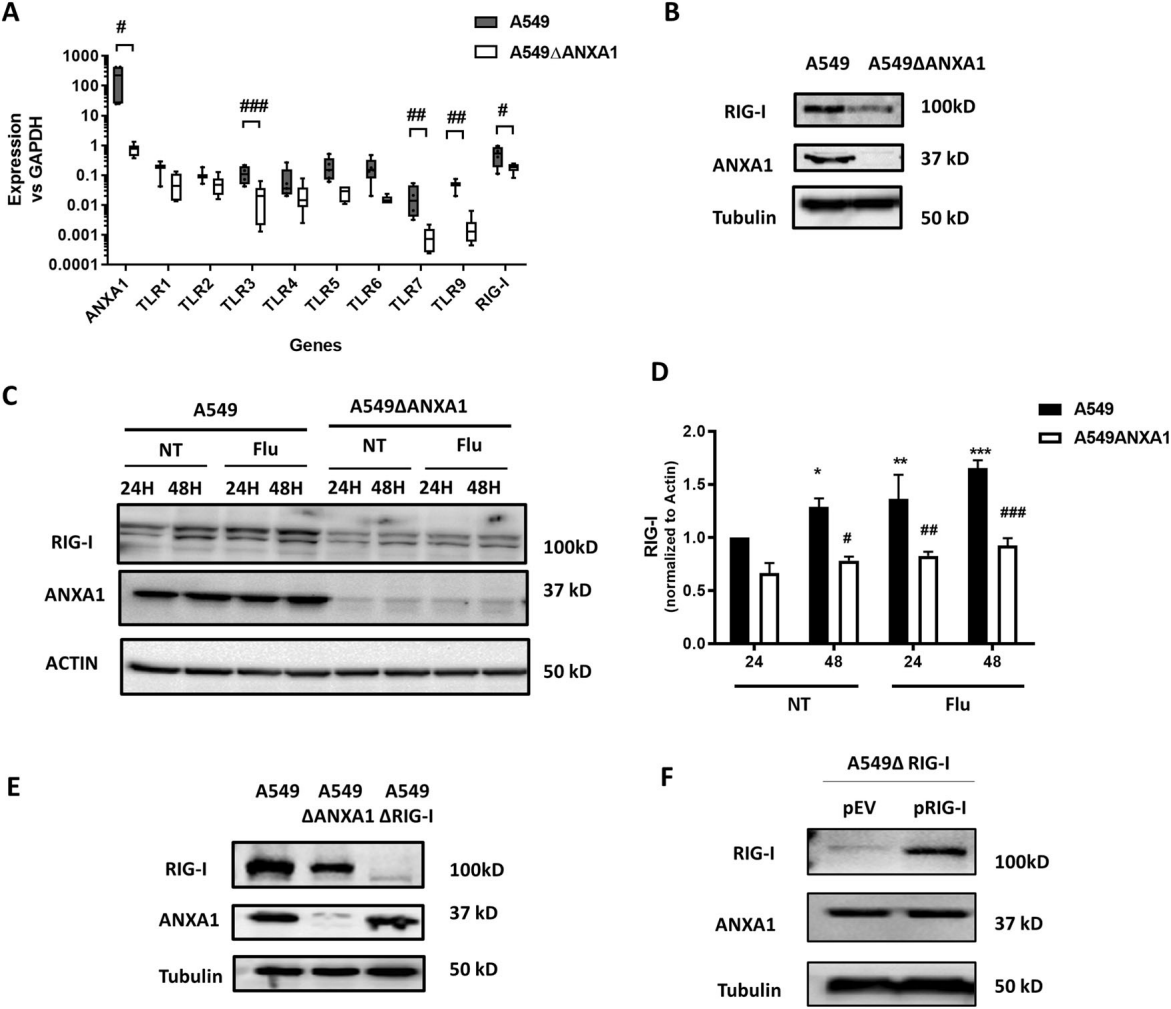
Lower expression of pattern recognition receptors in the absence of ANXA1 in A549 lung epithelial cells. **a** Basal expression levels of ANXA1, TLRs, and RIG-I were determined using real-time PCR. mRNA expression is normalized to GAPDH internal control and is represented as mean ± SEM of *n* = 3 independent experiments. *P < 0.05; **P < 0.01; ***P < 0.001 vs. controls, #*P* < 0.05; ##*P* < 0.01; ###*P* < 0.001 vs. A549 parental cells. **b**, **e** Protein expression of ANXA1 and RIG-I was analyzed by western blotting. Protein levels of RIG-I and ANXA1 were analyzed **c** after influenza A/PR8 infection at indicated time points and cell lines and **d** quantified using Image-J analysis software, normalized to actin. **f** RIG-I was re-expressed in A549∆RIG-I cells. Representative images are displayed.

We first investigated the role of ANXA1 in regulation of the expression of anti-microbial pattern recognition receptors (PRR) using the A549 and A549∆ANXA1 cells. Figure 1a shows that TLR3, TLR7, and TLR9 were significantly down-regulated in A549∆ANXA1 compared to A549 basally. In addition, the basal gene expression of RIG-I was similarly down-regulated in A549∆ANXA1 relative to A549 (Fig. 1a). Protein expression of RIG-I is also lower in cells with deleted ANXA1 (Fig. 1b) suggesting that ANXA1 may play a regulatory role in the expression of RIG-I basally.

Levels of RIG-I were found to be up-regulated by H5N1 virus A/Vietnam/1203/04 in ducks due to a positive feedback loop to amplify anti-viral responses^16^. Therefore, cells were infected with IAV PR8 with M.O.I. of 1 for 24 and 48 h using RIG-I levels as a read-out. A significant increase in RIG-I was observed when A549 cells but not A549 ∆ANXA1 cells treated with IAV infection. This suggests that in the context of IAV infection, the RIG-I signaling pathway is impaired when A549 epithelial cells are deficient in ANXA1 (Fig. 1c, d).

We proceeded to generate a RIG-I knock out A549 cell line using CRISPR-Cas9 technology (Supplementary Fig. 2A). To confirm that RIG-I has been successfully deleted in A549∆RIG-I cell line, Sanger sequencing was performed. The guide RNA, sgRNA #4, targets the part of the RIG-I gene encoding for the CARD domain (Supplementary Fig. 2B). The sequence targeted by sgRNA #4 in the RIG-I gene was amplified using PCR and the product from Sanger sequencing revealed a 72 base pair deletion in the CARD domain (Supplementary Fig. 2C). Furthermore, to ensure that there are no off-target effects, an online tool COSMID was also used which suggests two possible off target effects in HHIP-ASI allowing for two base pair mismatches and RAB11 FIP5 with one base pair mismatch (data not included).

In A549∆RIG-I cells, the basal level of ANXA1 did not change compared to A549 cells (Fig. 1e). In addition, the re-expression of RIG-I via transient transfection did not affect the levels of ANXA1 (Fig. 1f).

**Fig. 2:**
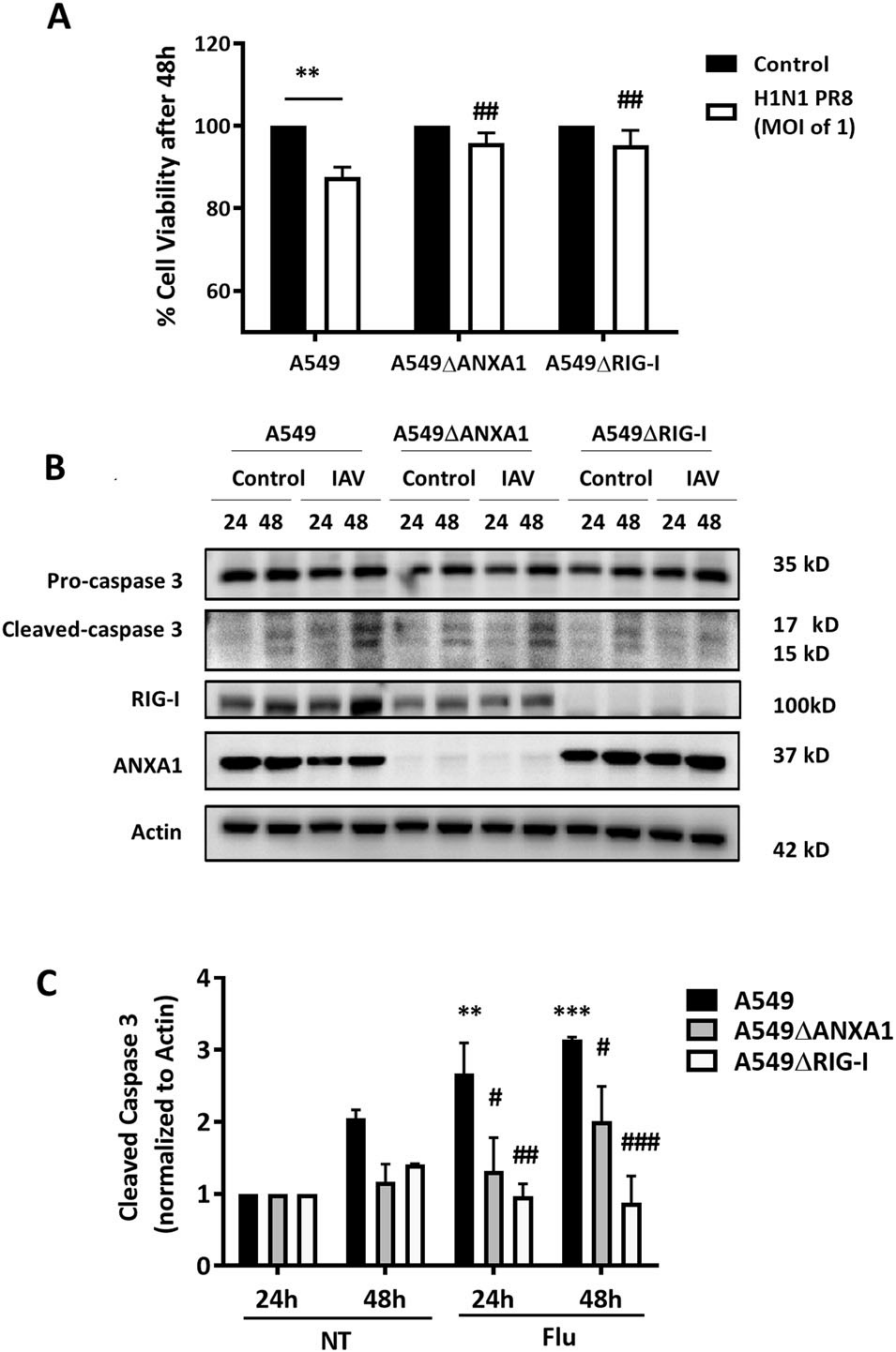
Absence of ANXA1 and RIG-I abrogates the reduction of cell viability in H1N1 infection of A549 cells. Cells were infected with 1 MOI H1N1 PR8 for the stated times. **a** Cell viability was determined using Cell Titer assay. Absorbance was read at 490 nm 2 h after addition of cell Titer substrate. **b** Lysates were collected at stated time points and assayed for the indicated proteins. Blots are representative of three independent experiments. **c** Cleaved caspase 3 (both 15 and 17 kDa) was quantified and normalized to actin. Data is represented as mean ± SEM of *n* = 3 independent experiments. *P < 0.05; **P < 0.01; ***P < 0.001 vs. controls, #*P* < 0.05; ##*P* < 0.01; ###*P* < 0.001 vs. A549 parental cells using two-way ANOVA and Bonferonni post-tests.

Next, to investigate the role of ANXA1 and RIG-I in IAV-induced cell death, A549, A549∆ANXA1, and A549 ∆RIG-I cells were infected with 1 multiplicity of infection (MOI, i.e., 1 viral particle per cell) H1N1 PR8 virus for 24 and 48 h. A549 cells infected with IAV exhibited significant reduction in cell viability while no significant change in cell viability was observed with IAV-infected A549∆ANXA1 or A549∆RIG-I cells (Fig. 2a). Similarly, a significant increase in cleaved caspase 3 levels was observed in IAV-infected A549 cells when compared to A549∆ANXA1 and A549∆RIG-I infected cells (Fig. 2b, c).

**Fig. 3:**
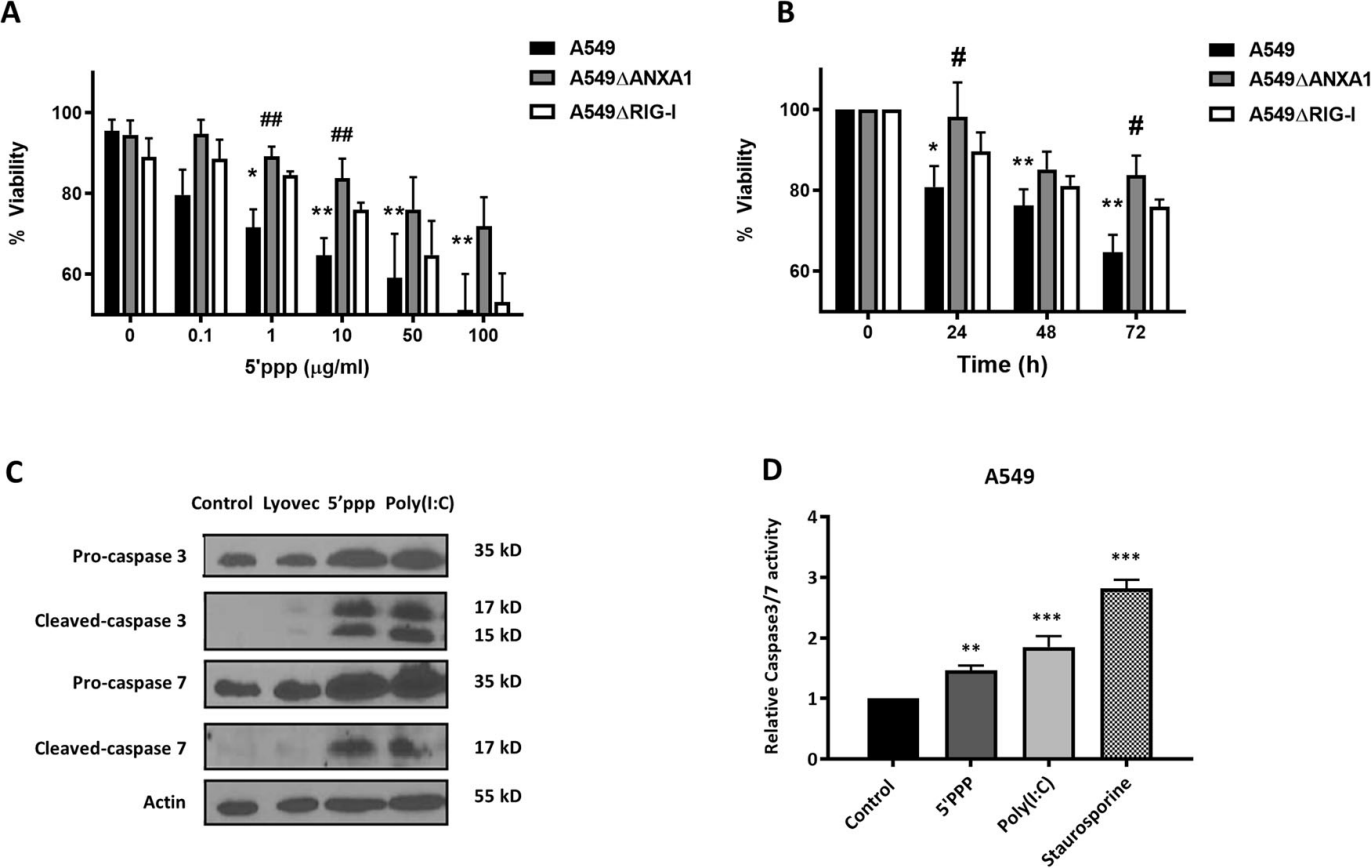
Absence of ANXA1 inhbits the reduction of cell viability after 5′ppp-RNA activation of RIG-I. Cells were transfected with Lyovec control or stated concentrations of 5′ppp-RNA with Lyovec. **a** Cell viability was determined using Cell Titer assay and normalized to the Lyovec control after 48 h with the indicated concentrations of 5’ppp-RNA or **b** at the stated times after transfection with 10 μg/ml of 5′ppp-RNA. Absorbance was read at 490 nm 2 h after addition of cell Titer substrate. **c** Lysates were collected at 48 h after 5′ppp-RNA transfection and assayed for the indicated proteins. Blots are representative of three independent experiments. **d** Caspase 3/7 activity was quantified using caspase3/7 Glo (Promega). Data is represented as mean ± SEM of *n* = 3–4 independent experiments. *P < 0.05; **P < 0.01; ***P < 0.001 vs. controls, #*P* < 0.05; ##*P* < 0.01 vs. A549 parental cells using two-way ANOVA and Bonferonni post-tests.

As IAV can trigger multiple RNA sensors in addition to RIG-I, and to better and more clearly elucidate the role of RIG-I signaling A549, A549∆ANXA1, and A549 ∆RIG-I cells were transfected with 5′ppp-RNA, a synthetic RIG-I-specific agonist for increasing time points^17^. Transfection of 1–500 µg/ml of 5′ppp-RNA transfection resulted in a significant reduction in cell viability in A549 lung epithelial cells, which was not observed in A549 ∆RIG-I cells, except for the highest concentration, confirming the specificity of 5′ppp-RNA to stimulate RIG-I (Fig. 3a). Interestingly, RIG-I stimulation with 5′ppp-RNA did not significantly reduce cell viability in A549∆ANXA1 cells, indicating that ANXA1 is required for RIG-I-induced cell death (Fig. 3a). Again, a time-dependent reduction in cell viability was observed with 5′ppp-RNA stimulation, which was inhibited with the absence of ANXA1 and RIG-I (Fig. 3b). Caspase 3 and caspase 7 cleavage and activity were markedly increased in A549 cells after stimulation with 1 μg/ml 5′ppp-RNA after 48 h (Fig. 3c). 5′ppp-RNA, as well as transfected poly(I:C), another activator of RIG-I significantly increased caspase 3/7 activity (Fig. 3d).

**Fig. 4:**
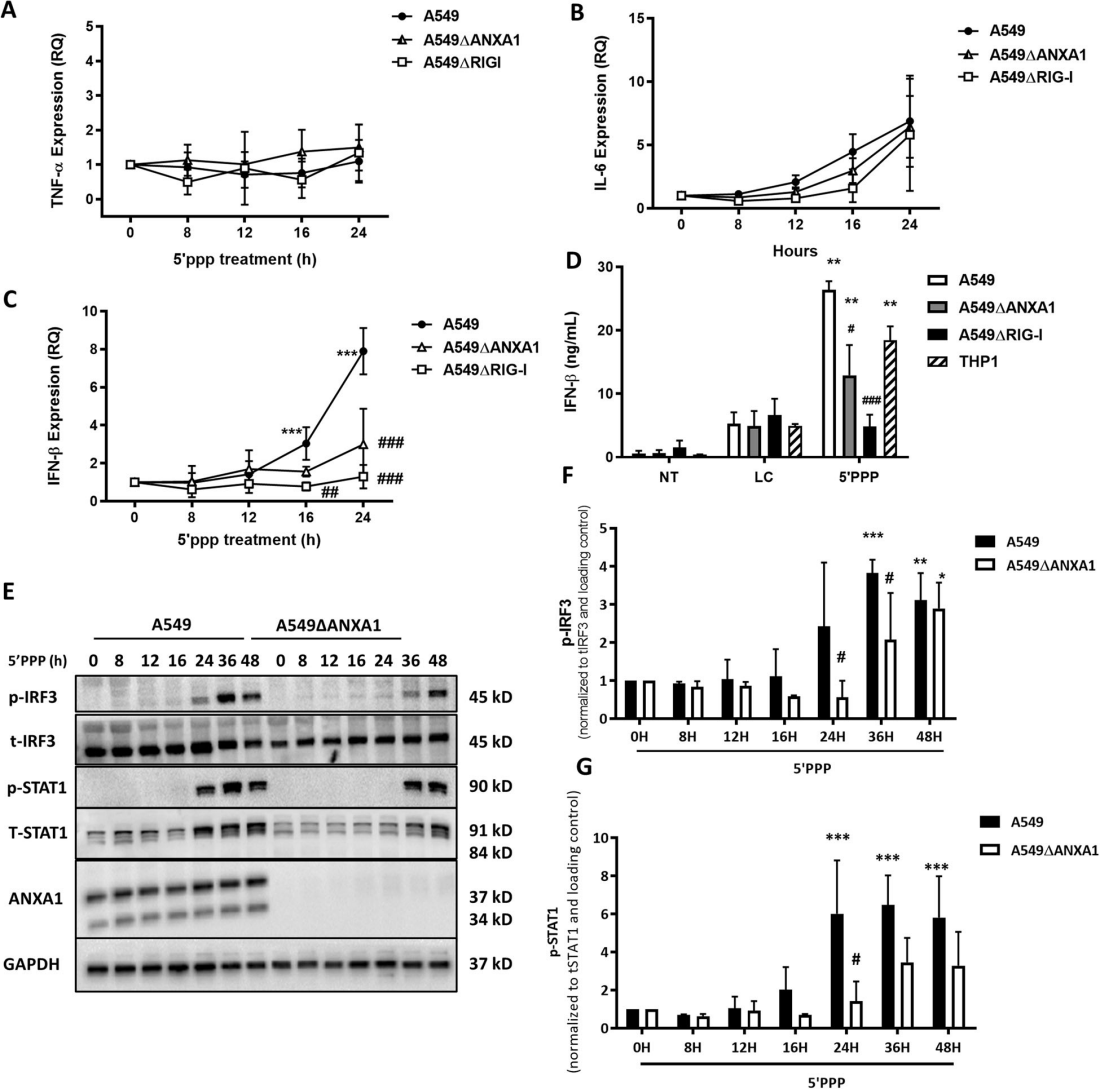
ANXA1 promotes IFNβ production and more rapid IRF3 activation after RIG-I stimulation. Cells were transfected with Lyovec control or 1 μg/ml of 5′ppp-RNA with Lyovec. **a**–**c** TNF-α, IL6, and IFNβ expression was measured with quantitative real-time PCR after the indicated times. **d** IFNβ production was measured using ELISA after 48 h. **e** Lysates were collected at indicated times after 5′ppp-RNA transfection and assayed for the indicated proteins. Blots are representative of three independent experiments. **e**, **f** Phosho-IRF3 or STAT1 was quantified and normalized to their respective total proteins and actin. Data is represented as mean ± SEM of *n* = 4 independent experiments. *P < 0.05; **P < 0.01; ***P < 0.001 vs. controls, #*P* < 0.05; ##*P* < 0.01; ###*P* < 0.001 vs. A549 parental cells using two-way ANOVA and Bonferonni post-tests.

Next, we determined if the pro-inflammatory cytokines TNF-α and IL-6 play a role in apoptosis induced by RIG-I activation, TNF-α and IL-6 expression was determined by quantitative PCR. Upon transfection of 5′ppp-RNA for increasing time points, no increase in TNF-α mRNA was observed up till 24 h (Fig. 4a). IL-6 expression increased at 24 h, but was not significantly different when ANXA1 or RIG-I were deleted (Fig. 4b), indicating that the expression of IL-6 was non-specific to RIG-I. Next, to elucidate if the Type I IFN response was involved in inducing cell death in A549 lung epithelial cells, IFN-β expression was measured for 24 h. As shown in Fig. 4c, IFN-β expression was increased at 16 and 24 h, which was significantly abrogated when ANXA1 or RIG-I were deleted. Next, the levels of secreted IFN-β was measured using ELISA, and a significant production of IFN-β was observed after 48 h of treatment with 5′ppp-RNA (Fig. 4d). This production of IFN-β was inhibited in A549∆ANXA1 cells, and more so in A549∆RIG-I cells, suggesting a role for ANXA1 in the production of IFN-β, and the specificity of 5′ppp-RNA, respectively. The production of IFN-β was also measured in THP1 monocytic cells, which was comparable to A549 cells, indicating the capability of lung epithelial cells to be producers of IFN-β during IAV infection at late time points.

Next, we investigated the kinetics of RIG-I activation and the role of ANXA1. Unlike innate immune cells, which rapidly phosphorylate IRF3 after TLR or RIG-I stimulation, A549 lung epithelial cells exhibit a marked phosphorylation of IRF3 only after 24 h, peaking at 36 h. The kinetics of RIG-I activation is delayed in A549∆ANXA1 cells, with a peak only at 48 h post-stimulation (Fig. 4e), with a significantly lower p-IRF3 in A549∆ANXA1-treated cells at 24 and 36 h (Fig. 4f). This delayed activation of IRF3 could explain the lower levels of IFN-β secreted in A549∆ANXA1 cells. A delay in the phosphorylation of STAT1 in A549∆ANXA1-transfected cells was also observed which occured 36 h post-transfection of 5′ppp-RNA compared to 24 h post-transfection in A549 cells (Fig. 4e, g). This data shows that ANXA1 enhances IRF3 activation to modulate STAT1 signaling axis in RIG-I-activated lung epithelial cells.

**Fig. 5:**
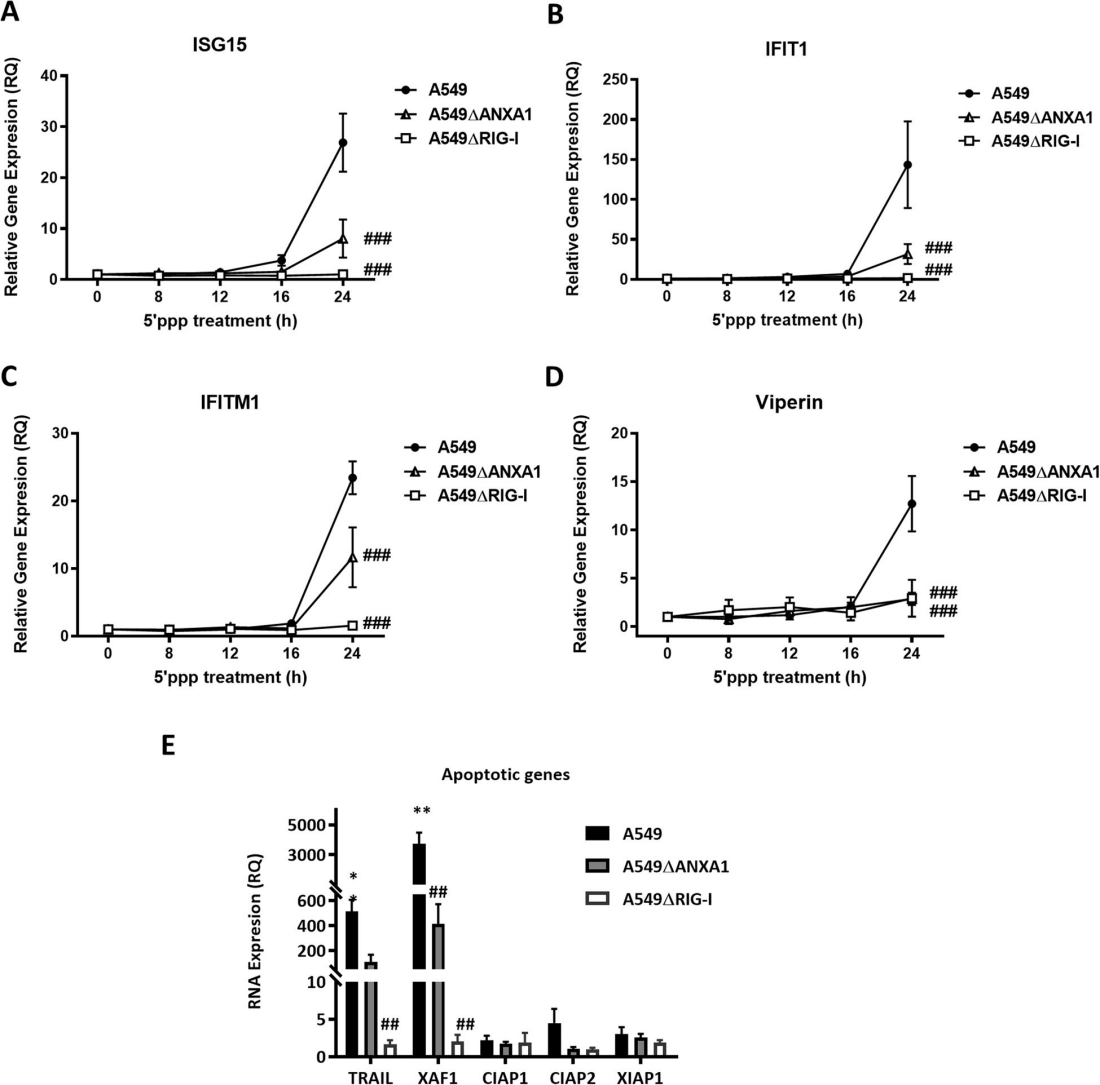
ANXA1 is partially required for the expression of interferon stimulated genes (ISGs) and apoptotic genes after RIG-I stimulation. Cells were transfected with Lyovec control or 1 μg/ml of 5’ppp-RNA with Lyovec. **a**–**d** ISG15, IFIT1, IFITM1, and Viperin expression was measured with quantitative real-time PCR after the indicated times. **e** Apoptotic genes were measured with quantitative real-time PCR after 48 h. Data is represented as mean ± SEM of *n* = 3 independent experiments. *P < 0.05; **P < 0.01; ***P < 0.001 vs. controls, ##*P* < 0.01, ###*P* *<* 0.001 vs. A549 parental cells using two-way ANOVA and Bonferonni post-tests.

To determine if RIG-I activation by 5′ppp-RNA results in the upregulation of anti-viral ISGs, and if ANXA1 is involved in ISG expression, *Isg15, USP18, Ifit1, Ifitm1*, and *Viperin* was measured. After 5′ppp-RNA transfection, *Isg15, USP18, Ifit1, Ifitm1*, and *Viperin* were all increased in A549 parental cells, but significantly less in A549∆ANXA1 5′ppp-RNA-treated cells, albeit still expressed for *Isg15, USP18, Ifit1, Ifitm1* when compared to A549∆RIG-I cells (Fig. 5a–c). Interestingly, no expression of *Viperin* was observed in A549∆ANXA1 5′ppp-RNA transfected cells, suggesting that ANXA1 may play an especially critical role in the expression of *Viperin* in A549 RIG-I-activated cells (Fig. 5d). In addition, to examine if RIG-I activation can stimulate the expression of pro-apoptotic genes to enhance the apoptotic process, the expression of pro-apoptotic genes (*Trail, XAF1*) as well as anti-apoptotic genes (*Ciap1, Ciap2, XIAP1*) were measured after 48 h of 5′ppp-RNA stimulation. *Trail* and *XAF1* were highly expressed in parental A549 cells, lower in A549∆ANXA1 5′ppp-RNA transfected cells, and not expressed in A549∆RIG-I 5′ppp-RNA transfected cells, indicating that ANXA1 is only partially involved in the upregulation of these pro-apoptotic genes.

**Fig. 6:**
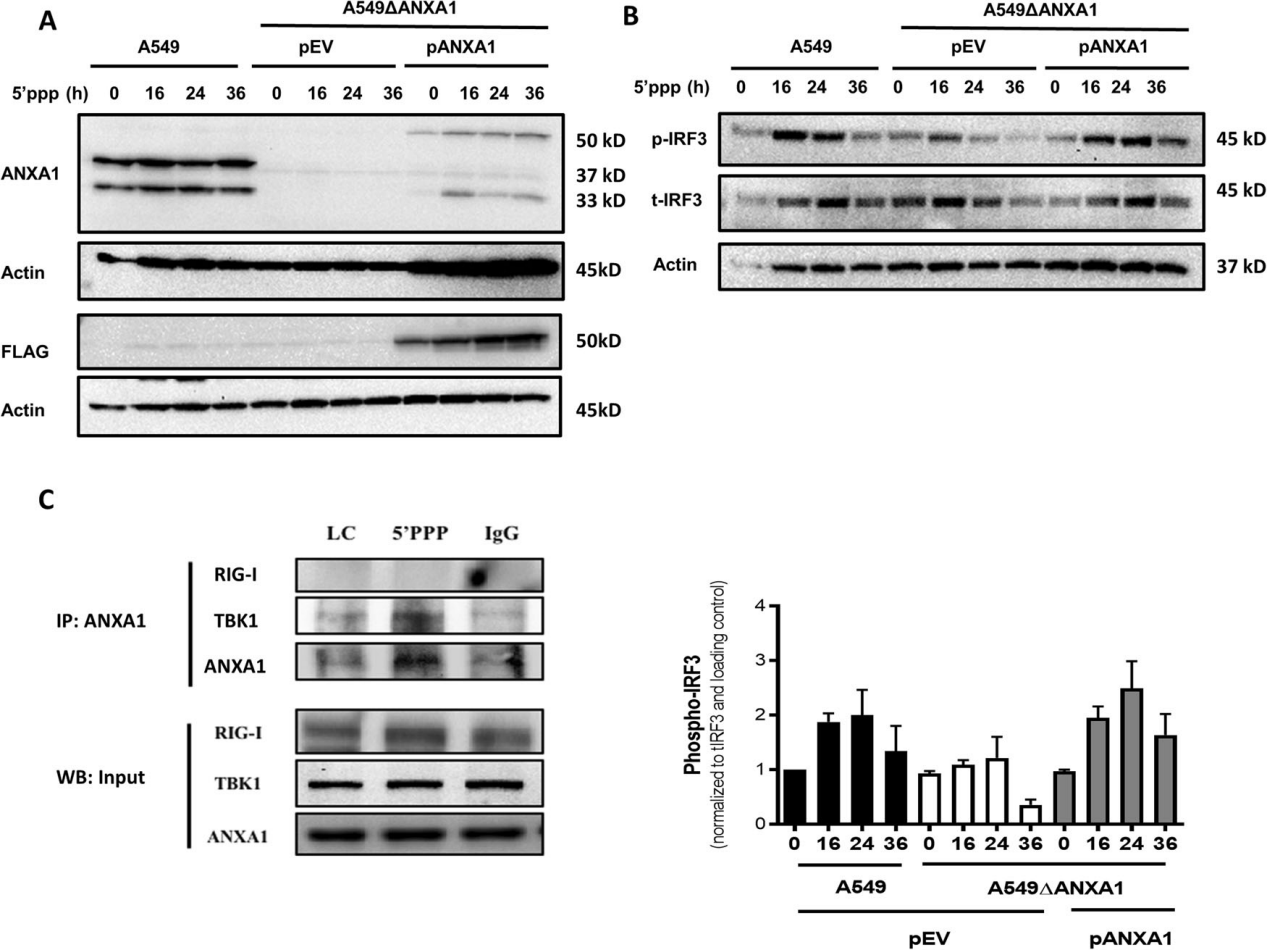
Re-expression of ANXA1 in A549∆ANXA1 restored IRF3 activation when RIG-I is activated. Western blot of ANXA1 in A549 and A549∆ANXA1 cells transfected with pEV (pCMV10-3xFLAG) or pANXA1 (pCMV10-3xFLAG-ANXA1) for 24 h before transfection with 1 μg/ml of 5′ppp-RNA. Proteins that were probed were **a** ANXA1 and **b** p-IRF3 and T-IRF3, respectively. Actin was used as protein loading control. Densitometry analysis of p-IRF3 and total IRF3 levels normalized to protein loading control. Data is represented as mean ± SEM of *n* = 3 independent experiments. **c** Immunoprecipitation of A549 treated with Lyovec and 5′PPP after 20 h using anti-ANXA1 antibody for pulldown and probed with RIG-I, TBK1, and ANXA1 levels.

To confirm that ANXA1 plays a critical role in the signaling kinetics of RIG-I activation, we re-expressed ANXA1 back into A549ΔANXA1 cells using a pCMV10 plasmid with 3xFLAG tag encoding human ANXA1 protein (pANXA1). As controls, cells were also transfected with a control empty vector plasmid (pEV). The over-expression of ANXA1 was confirmed where the ANXA1-3xFLAG band was observed at a higher molecular weight of ~50 kDa compared to endogenous ANXA1 at 37 kDa. As can be seen in Fig. 6a, ANXA1 was expressed as full length and cleaved proteins in both A549 and pANXA1 overexpressed cells. After 5′ppp treatment, IRF3 phosphorylation was observed to be lower in A549∆ANXA1 cells. However, when ANXA1 was re-expressed into A549∆ANXA1, the phosphorylation of IRF3 was restored to the levels observed in A549-treated cells (Fig. 6b). Thus, this data confirms our hypothesis that ANXA1 plays a role in RIG-I-activated IRF3/STAT1 signaling in A549 lung epithelial cells where an absence results in dampened IRF3 activation.

To investigate how ANXA1 affects the IRF3/STAT1 signaling axis upon RIG-I activation, an immunoprecipitation assay was conducted where ANXA1 was pulled down and probed with various proteins in the IRF3 pathway. Figure 6c shows that ANXA1 does not bind to RIG-I when RIG-I is activated with 5′PPP. Previously however, we have shown that ANXA1 physically associates with TBK1 basally and continues to be associated when TLR4 is activated with lipopolysaccharide treatment^18^. TBK1 is upstream of IRF3 where the activation of TBK1 results in the activation of IRF3. Thus, the association of ANXA1 with TBK1 was investigated after A549 cells were transfected with 5′PPP. When RIG-I is activated by 5′PPP in A549 cells, TBK1 is similarly found to associate with ANXA1. Thus, we propose that when RIG-I is activated, ANXA1 regulates IRF3 activity by its association to TBK1.

**Fig. 7:**
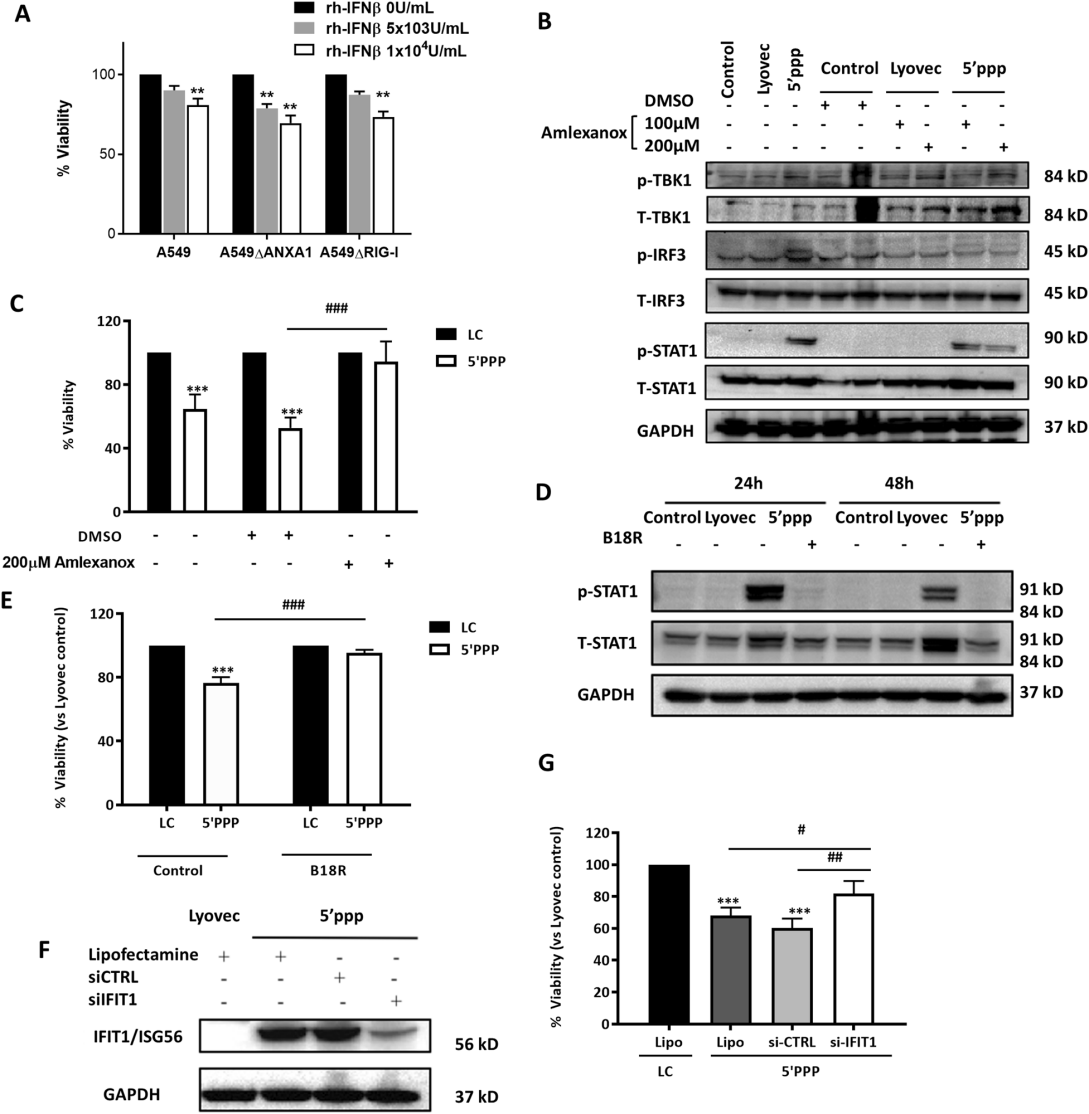
5′ppp-RNA activation of RIG-I stimulates the IRF3–IFNβ–IFNAR–STAT1–IFIT1 pathway to induce cell death of A549 cells. Cells were transfected with Lyovec control or 1 μg/ml of 5′ppp-RNA with Lyovec. **b**, **d**, **f** Lysates were collected at indicated times after 5′ppp-RNA transfection and assayed for the indicated proteins. Blots are representative of three independent experiments. **a**, **c**, **e**, **g** Cell viability was determined using Cell Titer assay and normalized to the Lyovec control after 72 h. Specifically, cells were **a** treated with human recombinant IFNβ for the stated times, **b**, **c** pre-and cotreated with 100 or 200 μM Amlexanox, a TBK1 antagonist 1 h prior to 5′ppp-RNA transfection, **d**, **e** pre-and co-treated with 0.1 μg/ml B18R an IFNAR antagonist 1 h prior to 5′ppp-RNA transfection, and **f**, **g** transfected with negative siCTRL and siIFIT1 16 h post-transfection of 5′ppp-RNA. Data is represented as mean ± SEM of *n* = 3 independent experiments. *P < 0.05; **P < 0.01; ***P < 0.001 vs. controls, #*P* < 0.05; ##*P* < 0.01; ###*P* < 0.001 vs. A549 parental cells using two-way ANOVA and Bonferonni post-tests.

To determine if ANXA1 expression only regulates the first phase of RIG-I activation (IRF3 phosphorylation and IFNβ production), or if it also regulates the second phase (IFNRA binding to IFNβ and STAT1 activation), we treated A549 cells with recombinant human IFN-β and analyzed cell viability over time. With 1 × 10^4^ U/ml of rh-IFN-β treatment, a significant reduction in proliferation after 72 h was observed in all the cell lines, with and without ANXA1 or RIG-I, suggesting that IFN-β production was required for the reduction in cell proliferation observed, and that ANXA1 acted upstream of IFN-β (Fig. 7a). In Sendai virus infection of A549 epithelial cells, it was reported that the induction of apoptotsis was independent of IRF3 transcriptional activity^19^. Thus to investigate if IRF3 transcriptional activity is needed for RIG-I-induced apoptosis, we first inhibited TBK1 signaling using a selective TBK1 inhibitor, Amlexanox. Co-treatment of 200 μM Amlexanox with 5′ppp-RNA inhibited IRF3 and STAT1 activation (Fig. 7b), as well as the 5′ppp-RNA-induced cell death (Fig. 7c). Next, to determine if IFN-β association with the IFNAR receptor was required, B18R, an IFNAR antagonist was used, and pretreatment of cells with B18R prior to 5′ppp-RNA stimulation reversed the cell death induced by 5′ppp-RNA (Fig. 7d), as well as an inhibition of STAT1 activation after 24 and 48 h (Fig. 7e), indicating a requirement for the IFNAR and STAT1 in this pathway. This clearly demonstrates that the transcriptional activity of IRF3 to induce production of IFN-β, leading to the association with IFNAR and downstream STAT1 activation is necessary for the induction of RIG-I-induced cell death.

We predicted that ISG56/IFIT1 may mediate the induction of apoptotic cell death upon RIG-I activation. Thus, we silenced ISG56/IFIT1 using siRNA 16 h post 5′ppp-RNA transfection of A549 cells. When ISG56/IFIT1 was successfully silenced (Fig. 6f), there was a partial rescue in cell death induced by 5′ppp-RNA transfection of A549 cells after 72 h (Fig. 6g). This data suggests that cell death due to type I IFN response may be mediated by the up-regulation of ISG56/IFIT1.

## Discussion

As ANXA1 may play a role in regulating multiple different pathways in IAV-infected A549 lung epithelial cells, the focus of this study was on the cytoplasmic RNA sensor RIG-I. A general screen of basal expression PRRs between A549 and A549∆ANXA1 showed a lower expression of RIG-I. The expression of other RNA-associated PRRs like TLR3, TLR7, and TLR9 were also significantly lower in A549∆ANXA1 cells. In the context of IAV-infection, TLR7 and RIG-I are known to be activated^20–23^. This was confirmed when IAV infection of A549 epithelial cells resulted in an increase in the levels of RIG-I, possibly as part of a positive feedback loop to trigger greater anti-viral responses.

Previous reports have shown that activation of RIG-I in both immune and epithelial cells can induce cell death via apoptosis although the mechanism by which apoptosis is induced seems to differ between cell types and triggers. RIG-I activation due to Sendai virus and VSV infection was reported to activate TBK1/IKKε which in turn triggers the degradation of the anti-apoptotic protein, XIAP thus inducing apoptosis^24^. This was similarly reported for Vaccinia virus infection where RIG-I and MDA-5 activation induced apoptotic cell death was dependent on Type I IFN response that results in the induction of pro-apoptotic protein, NOXA^25^. In contrast, Sendai virus-induced apoptosis was dependent only on IRF3 activation but was independent of its transcriptional activity^19^. Instead it was proposed that the pro-apoptotic property of IRF3 is due to its ability to bind to pro-apoptotic protein Bax at the mitochondria to trigger mitochondrial-dependent apoptosis^26^.

With a clear dampening of Type I IFN response when RIG-I-activated lung epithelial cells are deficient of ANXA1, the exact regulatory role that ANXA1 plays was unknown. In 5′ppp-RNA-transfected A549∆ANXA1cells, there was a delay in the activation of IRF3 and subsequently of STAT1. Thus, the presence of ANXA1 seems crucial for the activation of IRF3 when RIG-I is activated. This positive regulatory role of ANXA1 was confirmed when the activation of IRF3 was rescued with re-expression of ANXA1 in these cells. To discover how ANXA1 mediates this regulatory role, previous studies of the role of ANXA1 in TLR3-activated Type I IFN response hints of the possible role that ANXA1 may play in RIG-I-activated IFN signaling. With TLR3 or TLR4 activation, ANXA1 was found to bind to TBK1 with its C-terminus, thereby regulating IRF3 ability to stimulate downstream activation of Type I IFN production^27^. In addition, the ability of ANXA1 to mediate its effects in various contexts is found to be through binding and activating its receptor, FPR2. However, its binding to TBK1 and thus regulating the downstream signaling upon TLR3/4 activation was found to be independent of FPR2^27^. This suggests that the endogenous ANXA1 in a cell can mediate pro-inflammatory responses by its binding to TBK1. Furthermore, we have shown that ISG56/IFIT1 plays a role in the induction of apoptosis. Previous reports indicate that ISG56/IFIT1 is able to suppress proliferation and induce apoptosis^28^, and ISG54/IFIT2 binds to ISG56/IFIT1 and ISG60/IFIT3 to mediate mitochondrial apoptosis^29^. ISG56/IFIT1, belongs to a family of proteins called the IFN-induced protein with tetratricopeptide repeats and also has anti-viral functions. ISG56 has been found to inhibit the replication of DNA and RNA viruses and silencing of ISG56 in Hepatitis C infection results in enhancement of viral replication^30^. However, similar to ISG15, ISG56 has also been shown to exert a negative pressure on the anti-viral signaling responses by interrupting the interaction of MITA (STING) with VISA and/or TBK1 upon RIG-I activation^31^. IFITM1, a tight junction protein, has also been found to exert anti-viral properties by inhibiting the entry of Hepatitis C Virus^32^ and also inhibit Zika virus replication in the early stages of infection^33^. Viperin similarly inhibits the replication of both DNA and RNA viruses^34^. Thus, the up-regulation of these anti-viral ISGs serve two functions that ultimately benefits the host. In addition to inhibiting the entry and replication of viruses, these ISGs also serves to place a brake on the inflammatory response first activated by RIG-I signaling to prevent excessive inflammation that may be harmful.

Taken together, our results demonstrate the role of ANXA1 in IAV infection of A549 epithelial cells and more specifically, its role in RIG-I activation. When A549 cells are deficient in ANXA1, this results in a suppression of apoptotic cell death by activation of RIG-I. We show that when RIG-I is activated in A549 lung epithelial cells by 5′ppp-RNA, this leads to a stimulation of the IRF3–IFNAR–STAT1-signaling pathway, activating the transcription of ISGs, which can play a role to induce apoptotic cell death.

## Materials and methods

### Chemicals and Inhibitors

5′ppp-RNA (5′ppp-dsRNA) and poly(I:C) was obtained from Invivogen (CA, USA) and used at working concentration of 1 μg/ml and 10 ng/ml unless stated otherwise, respectively. Staurosporine and cisplatin were both obtained from Sigma Aldrich (MI, USA). Staurosporine was dissolved in DMSO and cisplatin was dissolved in F12K media before treatment. The inhibitors used in this study were Amlexanox (Invivogen, CA, USA), B18R and human recombinant IFN-β (Stemcell Technologies, Vancouver, Canada) and Z-vad-fmk (Merck, NJ, USA).

### Cell lines

Human lung epithelial lung cell line A549 (ATCC^®^ CCL-185™) and associated CRISPR-Cas9 knock out A549 clones were cultured in complete F-12K media (Gibco^®^). A549 cell line stably transfected with scrambled vector (SC) and shRNA specific to Annexin A1 (ANXA1) were also cultured in complete F-12K media. All cell lines were grown and incubated in a 37 °C humidified incubator with 5% CO_2_ (Thermo Scientific, Singapore).

### Generating CRISPR/Cas9 knock out cell lines

CRISPR/Cas9 technology was employed to generate two knock out cell lines from human lung epithelial cell line A549 (ATCC^®^ CCL-185™). A549∆ANXA1 cell line is knocked out for ANXA1 protein and A549∆RIG-I cell line is knocked out for RIG-I receptor. Plasmids were obtained from Horizon Discovery (Waterbeach, UK). A549 cells were seeded into 10 cm tissue culture dishes and transfected the following morning with 10 µg of plasmid using TurboFect transfection reagent (Thermo Fisher Scientific, MA, USA) according to manufacturer’s protocol. 16–24 h after transfection, cells were trypsinized using Accutase^®^ (BioWest, MO, USA). Trypsinized cells were neutralized with complete Ham’s F-12K media and centrifuged at 1000 rpm for 3 min at room temperature. Supernatant was aspirated and cell pellet was resuspended in 1 ml complete media. Cell suspension was passed through a cell strainer and pipetted into FACs tubes. Cells were then sorted using Beckman-Coulter Mo-Flo Legacy Cell Sorter for GFP-positive cells and seeded as single cells into 96-well plates. Cell clones with a knock-out of target gene were determined via western blotting and Sanger sequencing.

### Infection of cells with Influenza A (IAV) PR8

The day before IAV infection, A549 cells were seeded into tissue culture plates with complete F12K media. Before infection, media was aspirated and the monolayer is washed with 1X PBS and serum-free F12K media were added. Cells were infected with IAV PR8 virus with MOI 1 and placed in the incubator for 1 h. After 1 h, media was aspirated and replaced with complete F12K media.

### Caspase 3/7 Glo Assay

Caspase 3 and 7 activtity were measured with Caspase 3/7 Glo Assay systems (ProMega, WI, USA) according to manufacturer’s instructions.

### Cell Titer Glo Luminescent Cell Viability Assay

Cell viability was measured using CellTiter-Glo^®^ Luminescent Cell Viability Assay (Promega, WI, USA). A549 cells were seeded in 96-well plates the day before treatment. Before treatment, media is aspirated and changed with fresh complete F12K media. At the end of treatment, 20 μl of CellTiter-Glo^®^ Reagent was added per 100 μl media present in each well. The plate was left to incubate in the 37 °C humidified incubator for 2 h in the dark. Absorbance was measured at wavelength 490 nm using the plate reader (Tecan SPARK 10M).

### Western Blot

Cells are harvested and the concentration of cell lysates are determined using 1X Bradford’s Reagent (Bio-Rad Laboratories, CA, USA) and measured against protein Pierce™ Bovine Serum Albumin (BSA) standards (Thermo Fisher Scientific, MA, USA). Protein samples were prepared by added 5X Loading Dye with 2-Mercaptoethanol to the sample and boiled at 100 °C for 5 min. Equal amount of proteins was loaded per well within each gel run that can range between 20 and 50 μg. Samples were loaded onto SDS–PAGE gels and ran with 1X running buffer. Wet transfer was carried out using PVDF membranes (Bio-Rad Laboratories, CA, USA). Membranes were blocked using 3% BSA for 1 h with shaking on an orbital shaker at room temperature. Membranes were washed three times with 1X TBST (1X TBS and 0.1% Tween-20) for 10 min each before incubated with primary antibodies overnight at 4 °C. After overnight incubation, membranes were incubated with secondary antibodies for 1 h at room temperature. Membranes were probed for chemiluminescent signals using Amersham ECL Prime Western Blotting Detection Reagent (GE Healthcare Life Sciences, Singapore) with a GelDoc (Bio-rad Laboratories, CA, USA). Primary antibodies used were Actin (Santa Cruz, TX, US), IFIT1 (GeneTex, CA, USA), Tubulin (Sigma-Aldrich, MI, USA), Annexin-A1, Cleaved Caspase 3, Cleaved caspase 7, caspase 3, caspase 7, GAPDH, phospho and total IRF3, phospho and total TBK1, RIG-I, phospho, and total STAT1 (Cell signaling Technology, MA, USA).

### Real-time PCR

RNA extraction was carried out using the GeneJET RNA Purification Kit (Thermo Fisher Scientific, MA, USA) according to manufacturer’s instructions, after which, cDNA synthesis was performed. GoTaq^®^ qPCR Master Mix (ProMega, WI, USA) was used and a master mix was prepared with each reaction and quantitative real-time PCR was run on ABI 7500 real-time PCR system (Applied Biosystems, CA, USA). GAPDH was used as internal loading control and relative expression was calculated using ∆∆Ct approximation.

### Human IFN-ß ELISA

Supernatant collected from wells with treated cells were kept at −80 °C. Human IFN-ß ELISA kit (ELabscience, Wuhan, China) were used according to manufacturer’s instructions.

### Statistical analyses

Data is represented as mean ± SEM of *n* = 3 independent experiments. Statistical analysis conducted with two-way Anova and Bonferroni post-tests.

## Supplementary information

Supplementary Figures
