# Supplementary material for: Annexin-A1 promotes RIG-I-dependent signaling and apoptosis via regulation of the IRF3–IFNAR–STAT1–IFIT1 pathway in A549 lung epithelial cells
Source: Cell Death Dis. 2020 Jun 15;11(6):463. doi: 10.1038/s41419-020-2625-7 (PMC7295754; doi:10.1038/s41419-020-2625-7)
Supplement: Supplementary file 1 — Supplementary Figures [file 41419_2020_2625_MOESM1_ESM.pdf]

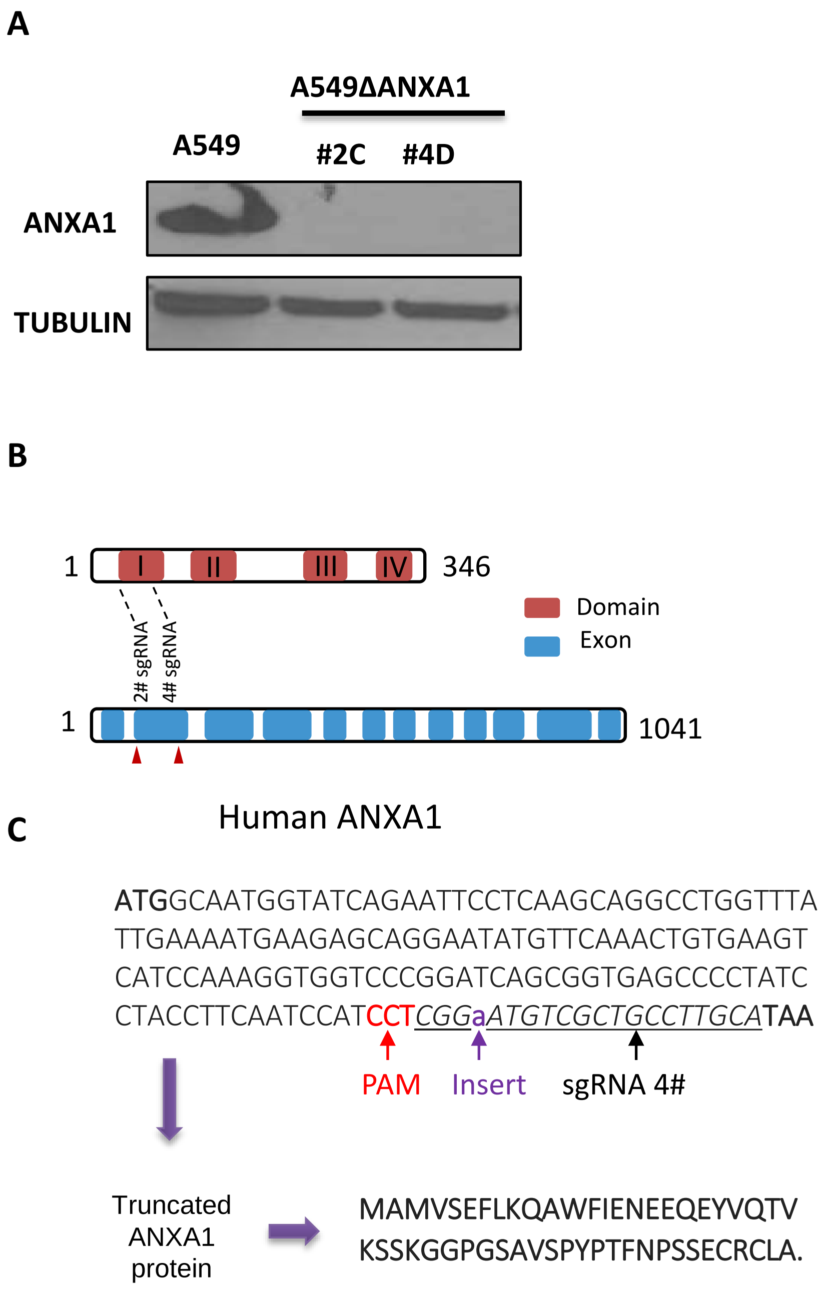

Supplementary Figure 1 . Crispr/Cas9 deletion of ANXA1 (A) ANXA1 was knocked out using Crispr/Cas9 technology and single clones were picked. Expression of ANXA1 determined using western blotting against ANXA1 protein. Actin is used as loading control. (B) Schematic representation of human ANXA1 protein with the respective locations sgRNA #2 and #4 were targeted. (C) Sequencing of A549 #4D clone via PCR revealed a successful insert of sgRNA #4 to result in the formation of a truncated ANXA1 protein. (B)

A

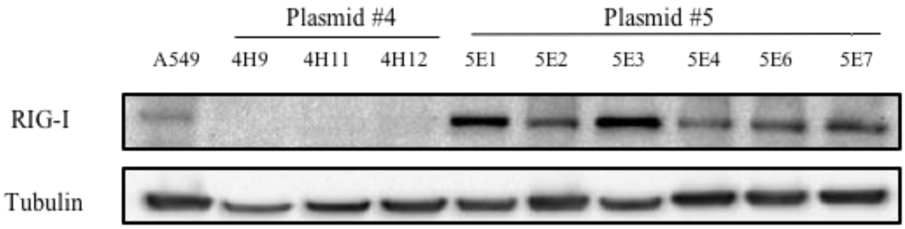

B

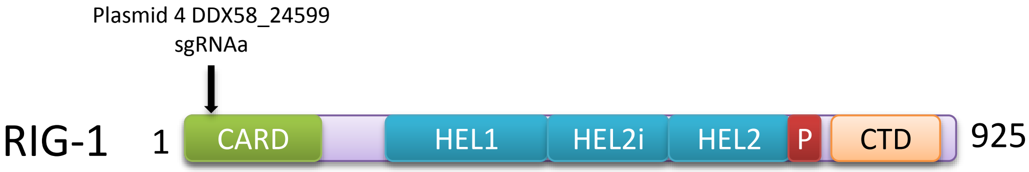

C

Sequencing forward primer

GGGAAACGAAACTAGCCCGAGGCAAAACAGCCTCCGCGAACCCCGCCCGCCGCTAGTT
GCACTTTCGATTTTCCCTTTAGTTATTAAAGTTCCTATGCAGCTCCGCCTCGCGTCCG
GCCTCATTTCCTCGGAAAATCCCTGCTTTCCCCGCTCGCCACGCCCTCCTCCTACCCG
GCTTTAAAGCTAGTGAGGCACAGCCTGCGGGGAACGTAGCTAGCTGCAAGCAGAGGCC
GGC**ATG**ACCACCGAGCAGCGACGCAG**CCT**GCAAGCCTTCCAGGATTATATCCGGAAGA

PAM sgRNA

72 bp deletion

CCCTGGACCCTACCTACATCCTGAGCTACATGGCCCCCTGGTTTAGGGAGGGTGAGTG
TCTCCAGCGGCGCCTTCTCGGCGGAAAACAATTGAGGACTTGTTTTTCTCTTGCTTCG
TTCCCTTCTTTTAATCAATGCAGAGAGCCTG***CCAGACTCCCCAGGTTTGTG***

Sequencing reverse primer

Supplementary Figure 2 . Crispr/Cas9 deletion of RIGI (A) RIG-I was knocked out using Crispr/Cas9 technology and single clones were picked. Expression of RIG-I determined using western blotting against RIG-I protein. Actin is used as loading control. (B) Schematic representation of human RIG-I protein with the respective locations sgRNA #4 was targeted. (C) Sequencing of A549 #4D clone via PCR revealed a successful insert of sgRNA #4 to result in 72 base pair deletion.
